# Burnout in Radiation Oncology Physician Workforce: The Effect of Mindfulness and Fulfillment

**DOI:** 10.1016/j.adro.2022.100971

**Published:** 2022-04-20

**Authors:** Jacob Eckstein, Zaker H. Rana, Sahar Caravan, Rajiv Sharma, Louis Potters, Bhupesh Parashar

**Affiliations:** Department of Radiation Medicine, Northwell Health Cancer Institute, Lake Success, New York

## Abstract

**Purpose:**

Mindfulness, defined as awareness of the moment while acknowledging and accepting one's feelings, thoughts, and sensations, is the aim of mindfulness meditation. Our objective was to investigate the relationship between burnout, mindfulness, fulfillment, and other personal characteristics in radiation oncology (RO) residents/attendings compared with other specialties.

**Methods and Materials:**

From December 2019 to February 2020, residents and attendings in multiple specialties at a single tertiary care academic institution were sent surveys, including the mindfulness attention awareness scale, Stanford professional fulfillment index, and a personal questionnaire. A Pearson correlation was conducted on the relationship between mindfulness, fulfillment, disengagement, and exhaustion. To determine risk factors for burnout (overall burnout ≥ 1.33), a univariate analysis was conducted to yield odds ratios (ORs) on debt, specialty, income, sleep, exercise, marital status, number of children, work hours, mindfulness (mindfulness attention awareness scale ≥ 4), fulfillment (professional fulfillment ≥ 3), and time with family/friends. Significant factors on univariate analysis were entered into multivariate analysis.

**Results:**

There were 180 surveys completed by 60 residents and attendings across 17 specialties. Eighteen (30%) respondents were in RO. Mindfulness positively correlated with fulfillment (*P* < .001, r = 0.534), negatively correlated with exhaustion (*P* < .001, r = -0.578), and negatively correlated with disengagement (*P* < .001, r = -0.483). Univariate analysis for factors associated with burnout was significant for mindfulness (OR = 0.065, *P* < .001), RO versus other specialty (OR = 0.024, *P* = .044), working >60 h/wk (OR = 5.091, *P* = .018), spending >10 h/wk with family or friends (OR = 0.120, *P* = .001), and fulfillment (OR = 0.103, *P* < .001). Multivariate analysis showed mindfulness and fulfillment to significantly decrease odds of burnout.

**Conclusions:**

RO physicians experienced less burnout than physicians in other specialties at our institution. Mindfulness, professional fulfillment, moderate work hours, and spending time with loved ones protected against burnout. Further study of interventions to promote mindfulness and fulfillment may help us understand how best to improve the mental and emotional health of RO physicians.

## Introduction

Despite a high level of compensation and professional prestige, physicians suffer higher rates of burnout, a work-related syndrome involving emotional exhaustion, depersonalization, and a sense of reduced personal accomplishment relative to the general population.^1^^,^^2^ Burnout correlates with numerous negative physician and patient outcomes, including self-perceived inferior clinical practice, medical errors, medical malpractice lawsuits, physician turnover, reduction of professional effort, physician depression, alcohol abuse, and suicidal ideation.^3^, ^4^, ^5^, ^6^ Appropriately, the scientific community has conducted investigations spanning multiple specialties to understand the causes of burnout, risk factors for physicians, and what measures can be taken to minimize its effect on both patient care and physician mental health.

One of the largest studies done on physician burnout to date surveyed over 5445 physicians in 2014, including 64 radiation oncology (RO) professionals, and found that RO professionals experienced the second lowest prevalence of burnout among all named specialties.^7^ When the investigators repeated the study in 2017, burnout in RO had increased relative to other specialties.^2^ In both of those studies, RO professionals also had higher burnout on average than the general population.^2^^,^^7^ Recently, there have been investigations to further characterize burnout and its risk factors among RO residents,^8^^,^^9^ program directors,^10^ and chairpersons.^11^ Some of the variables found to be significantly related to burnout in RO include work culture, work hours, nursing support, experience, debt, and interest in resilience training.^8^, ^9^, ^10^, ^11^

Although burnout is a vital measure of physician wellness and mental health, it fails to capture many positive indicators of healthy employment, including happiness at work, sense of meaning at work, and professional self-sufficiency. These elements were incorporated into a professional fulfillment assessment tool, the professional fulfillment index (PFI), which was experimentally validated to have a significant inverse correlation with burnout, depression symptoms, anxiety symptoms, and sleep-related impairment in a population of 250 physicians.^12^ Additionally, it was found to have strong sensitivity and specificity in detecting physicians with a “very good” quality of life.^12^

As we have learned that burnout and professional fulfillment are important and reliable indicators of physicians’ health, we have also learned that mindfulness can be protective against burnout. Mindfulness is defined as a nonjudgmental awareness and acceptance of one's physical sensations, feelings, and thoughts in the present moment. For example, while caring for an upset patient threatening a lawsuit, a mindful physician would be able to recognize his or her anxiety and frustration and accept it without excessive rumination, allowing focus on communication and patient care. Additionally, a mindful physician is able to live in the moment when relaxing after work, while a physician lacking mindfulness would be more likely to suffer from invasive concerns of malpractice litigation after hours. If follows that mindfulness is a resilience factor for burnout in primary care residents,^13^^,^^14^ and implementing mindfulness meditation has been shown to decrease burnout measures among practicing primary care physicians.^15^ After being shown to be a promising intervention in other specialties, mindfulness has become a part of many RO resident wellness programs,^9^ but it has not been directly studied in relation to burnout or fulfillment in the RO attending or resident population. Mindfulness is most commonly measured using the mindfulness attention and awareness scale (MAAS), which was validated to demonstrate strong psychometric properties, correlating with enhanced self-awareness, mindfulness mediation practice, behavior self-regulation, and positive emotional states in a landmark paper released in 2003.^16^^,^^17^ The scale has been validated in multiple languages across populations of students, physicians, and patients, including both meditators and nonmeditators.^16^, ^17^, ^18^, ^19^

In this study, we assessed burnout in a multidisciplinary population, including RO residents and attendings (30% of respondents) and residents in other specialties (70% of respondents) at a single institution. We also examined how burnout is related to mindfulness, fulfillment, specialty choice, and other lifestyle factors.

## Methods and Materials

### Survey distribution

After institutional review board approval, from December 2019 to February 2020, residents and attendings at a single tertiary care academic institution were sent surveys. Within the RO department, all residents (8) and attendings (19) were e-mailed individually. Outside of the RO department, surveys were distributed to 154 chief residents and fellows of other specialties with requests to forward the e-mail including the survey link to their respective programs’ trainees. Although all RO residents and attendings at our institution were sent surveys, the proportion of RO physicians targeted was limited given the large number trainees in other specialties at our institution. Survey Monkey (San Mateo, CA) was used to create the surveys, generate the sharable link, collect responses, and store the response data. Physicians within the RO department were also offered paper surveys to complete if preferred. No personal identifying information was collected, including internet protocol addresses of responders, which were blinded from the investigators. The e-mail included instructions to complete the survey only once, and a limit of 1 response per internet protocol address was set within the Survey Monkey settings. Two reminder emails were sent 1 week apart encouraging participation.

### Survey structure

The survey consisted of 17 questions and 3 sections. The first section was the MAAS,^16^ which accounted for 1 multipart question. The second section included questions about personal and lifestyle factors hypothesized to be related to fulfillment and accounted for questions 2 to 14. The third and final section consisted of the PFI^12^ separated into 3 multipart questions. The questions in each section and their purpose are described in the following sections.

### Mindfulness assessment

Section 1 included the MAAS,^16^ which is a 15-item instrument to assess mindfulness. Each item is scored from 1 to 6, and the scores are averaged to give a composite score, with higher scores implying more mindfulness. For this study, being mindful was defined as an MAAS ≥ 4.

### Lifestyle factors

Section 2 included questions about debt, salary (current salary for attendings and projected salary after graduation for residents and fellows), Post-graduation year (residents only), years of experience (attendings only), specialty, marital status, number of children, age, exercise frequency, work hours, and time with family and friends. The survey was distributed initially without the questions about work hours, free time with family and friends, and exercise habits. After these initial surveys were sent, the decision was made to add those questions because we hypothesized that burnout and satisfaction may be related to those factors. Thus, only non-RO physicians answered those questions.

### Assessment of burnout and fulfillment

Section 3 included the PFI, which is a validated 16-item instrument to assess physicians’ professional fulfillment and burnout.^12^ It assesses 3 categories of responses: fulfilment, interpersonal disengagement (ID), and work exhaustion (WE) using 5-point Likert scales (“not at all true” to “completely true” for professional fulfillment items and “not at all” to “extremely” for WE and ID items). All responses are scored 0 to 4. The professional fulfillment scale assesses the degree of intrinsic positive reward the individual derives from his or her work, including happiness, meaningfulness, contribution, self-worth, satisfaction, and feeling in control at work. The WE scale assesses exhaustion. The ID scale assesses empathy and connectedness with patients and colleagues. Responses in each category are averaged to give an overall score from 0 to 4, and the scores for ID and WE are averaged to give a score for overall burnout. Larger scores are positive for fulfillment and negative for WE and ID. Physicians were classified as experiencing burnout if they had an overall burnout score ≥ 1.33. Physicians were said to be professionally fulfilled if they had a fulfilment score ≥ 3, consistent with the cutoffs chosen when the index was validated.^12^

### Statistical analysis

Descriptive statistics were used to present survey respondent demographics. Continuous variables were expressed using sample medians and categorical variables as percentages. Pearson coefficient was used to examine correlation between mindfulness, fulfillment, exhaustion, disengagement, and burnout. Predictors of burnout were assessed by univariate and multivariate logistic regression. Results of univariate and multivariate logistic regression are reported as odds ratios (ORs) with 95% confidence intervals. A *P* value of < .05 was considered significant. Factors in univariate analysis with *P* < .10 were retained for multivariate analysis. All statistical analysis was performed using SPSS 21 (IBM, Armonk, NY).

## Results

### Respondent characteristics

Overall, 60 physicians from 17 different specialties responded to the survey. Respondents included 49 (82%) trainees and 11 (18%) attendings. Half of the respondents were single, 48% were either married or engaged, and 1 (2%) was divorced. A majority of the respondents (72%) did not have children. Only 3 specialties composed greater than 10% of respondents; those specialties were RO with 18 (30%), internal medicine with 10 (17%), and family medicine with 7 (12%). All of the attending respondents were in RO. Among trainee respondents, 7 (14%) were in RO, and 42 (86%) were in other specialties. By proportion, Post-graduation year 1, 2, 3, 4, 5, 6, 7, and >7 residents composed 12%, 10%, 22%, 27%, 12%, 6%, 4%, and 4% of responding trainees. Many attendings had a lot of work experience; 54% had worked for at least 21 years, and none had worked less than 6 years. Most respondents had unpaid educational debt (73%), and 29% had greater than $300,000 in outstanding debt. Respondents worked a median of 60 to 70 hours weekly, and 8% worked greater than 80 hours weekly. The median speculative income (residents)/actual income (attendings) was between $200,000 to $300,000 annually. The median respondent spent 6 hours sleeping per night, exercised 3 days per week, and spent between 6 to 10 hours weekly with friends and family. Respondent characteristics are summarized in Table 1.

**Table 1:** Descriptive data of respondent physicians

Characteristic	Respondents	%	n
Age, y: median, (range)	31, (25-70)		60
Position			60
Resident	49		
Appearing	11		
Postgraduate year (if resident)			49
1	6	12%	
2	5	10%	
3	11	22%	
4	13	27%	
5	6	12%	
6	3	6%	
7	2	4%	
>7	2	4%	
No response	2	4%	
Years of experience (if attending)			11
0-5	0	0%	
6-10	1	9%	
11-15	2	18%	
16-20	1	9%	
21-30	2	18%	
>30	4	36%	
No response	1	9%	
Hours worked per week			60
<40 hours	1	2%	
40 > 50 hours	9	21%	
50 > 60 hours	8	17%	
60 > 70 hours	11	27%	
70 > 80 hours	11	23%	
>80 hours	5	10%	
No response	15		
Total outstanding debt ($)			60
1-50,0000	6	10%	
50,001-100,000	2	3%	
100,001-200,000	8	13%	
200,001-300,000	10	17%	
>300,000	17	28%	
None	16	27%	
No response	1	2%	
Speculative attending income/income ($)			60
50,001-100,000	8	13%	
100,001-200,000	12	20%	
200,001-300,000	19	32%	
300,001-400,000	6	10%	
400,001-500,000	6	10%	
>500,000	9	15%	
Specialty			60
Cardiology	0	0%	
Dermatology	1	2%	
Diagnostic radiology	0	0%	
Emergency medicine	1	2%	
Family medicine	7	12%	
General surgery	4	7%	
Internal medicine	10	17%	
Interventional radiology	0	0%	
Medical oncology	2	3%	
Neurology	1	2%	
Neurosurgery	1	2%	
OB/GYN	4	7%	
Orthopedic surgery	0	0%	
Other	4	7%	
Otolaryngology	0	0%	
Pathology	1	2%	
Pediatrics	1	2%	
Physical Medicine and rehabilitation	1	2%	
Psychiatry	2	3%	
Pulmonology	1	2%	
Radiation oncology	18	30%	
Urology	1	2%	
Average sleep nightly (hours)			60
<5	2	3%	
6	28	47%	
7	22	37%	
8	4	7%	
>8	0	0%	
No response	4	7%	
Days per week with exercise			60
0	0	0%	
1	14	23%	
2	12	20%	
3	9	15%	
4	6	10%	
5	4	7%	
6	2	3%	
7	0	0%	
No response	13	22%	
Marital status			60
Single	30	50%	
Married	26	43%	
Engaged	3	5%	
Divorced	1	2%	
Number of children			60
0	43	72%	
1	7	12%	
2	8	13%	
3	2	3%	
>3	0	0%	
Time with friends and family per week			60
0-5 hours	13	22%	
6-10 hours	18	30%	
10-25 hours	8	13%	
25-35 hours	2	3%	
>35 hours	4	7%	
No response	15	25%	

*Abbreviations:* OB/GYN = obstetrics/gynecology; PM, physical medicine; R, rehabilitation.

### Mindfulness, burnout, and fulfillment

**Fig. 1:**
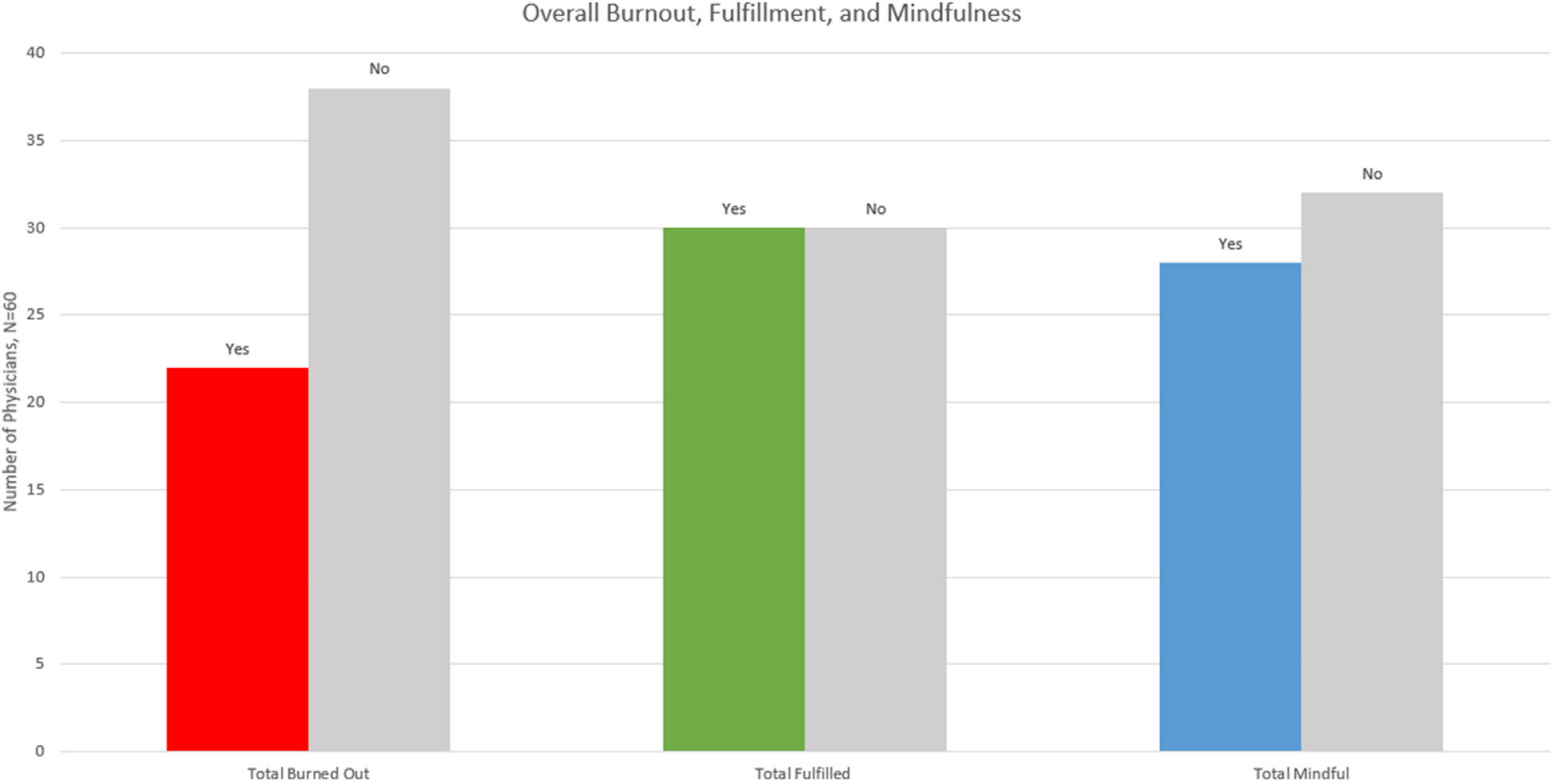
This figure shows the number of physicians who scored as mindful (>4 on mindfulness attention and awareness scale [MAAS]), fulfilled (≥3 on fulfillment portion of professional fulfillment index [PFI]), and burned out (≥1.33 on burnout portion of PFI).

The median MAAS score was 3.9 (range, 2.4-5.8) with a standard deviation of 0.89. Of the 60 respondents, 11 (18.3%) had a MAAS score of at least 5.0, 28 (47.7%) had a MAAS of at least 4.0, and 55 (91.7%) had a MAAS of at least 3.0, leaving only 5 (8.3%) with mindfulness less than 3.0. Nearly half (27/60) of physicians had MAAS scores within the range of 3.0 to 3.99. Median MAAS score was higher in RO compared with other specialties (4.27 vs 3.80). Median MAAS score was also higher among attending physicians compared with resident physicians (4.47 vs 3.80). Mindfulness of respondents is shown in Figure 1.

**Fig. 2:**
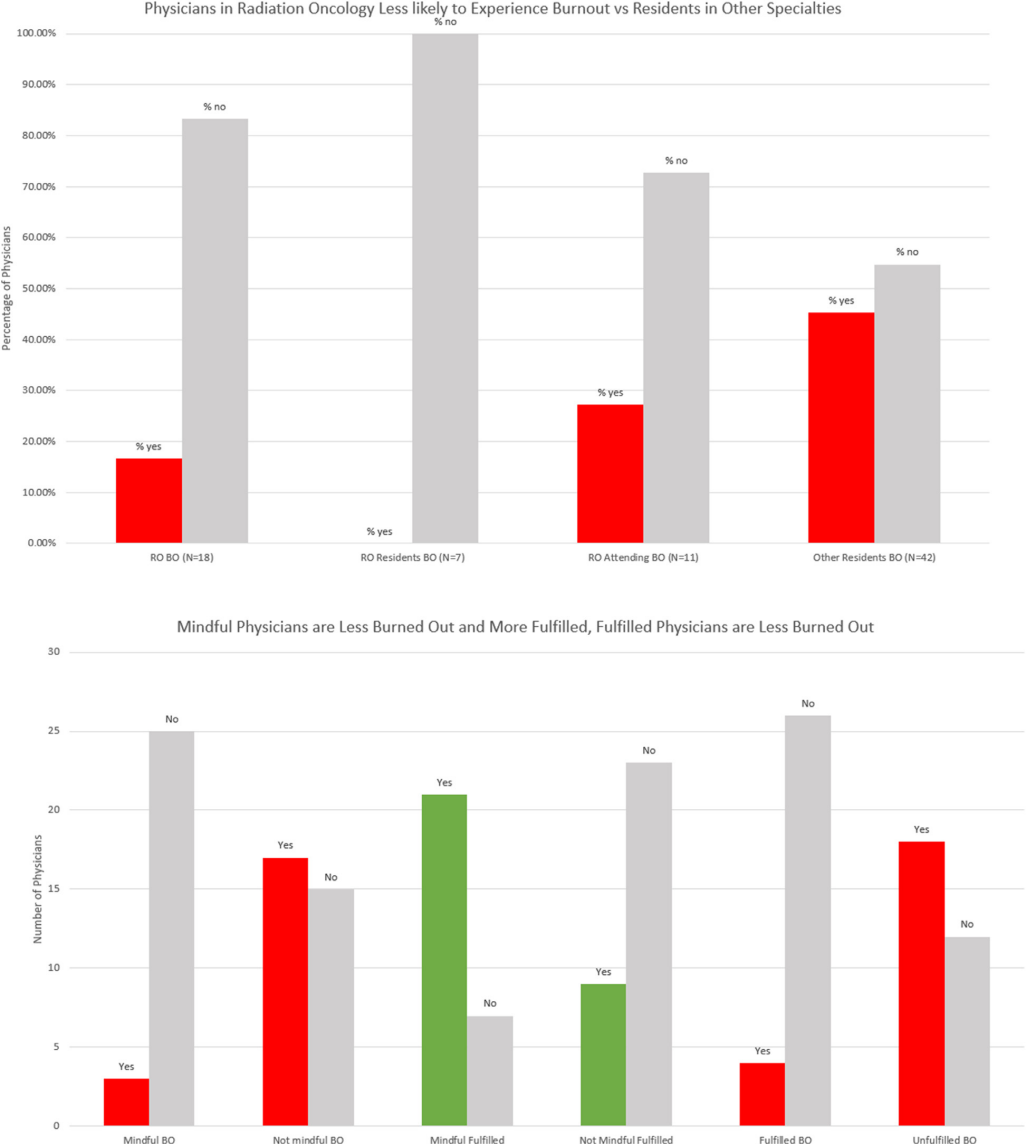
(a) This figure shows the percentage of physicians experiencing burnout among all radiation oncology physicians (RO BO), radiation oncology residents (RO residents BO), radiation oncology attendings (RO attendings BO), and residents in other specialties (other residents BO). (b) This figure shows that mindful physicians (based on mindfulness attention and awareness scale [MAAS] score ≥ 4) were less likely to experience burnout (score ≥ 1.33 on burnout portion of professional fulfillment index [PFI]) and more likely to experience fulfillment (score ≥ 3 on fulfillment portion of PFI) than those who were not mindful (MAAS score < 4). Finally, fulfilled physicians (≥3 on fulfillment portion of PFI) were less likely to experience burnout.

The median scores for the fulfillment, WE, and ID sections of the PFI were 2.92 (range, 0.83-4.00), 1.50 (range, 0.00-4.00), and 0.83 (range, 0.83-3.17), respectively, which resulted in a median overall burnout score of 1.00 (range, 0.00-3.58) with a standard deviation of 0.76. Overall, 23 (38%) respondents were burned out and 30 (50%) respondents were fulfilled based on PFI criteria. Median burnout score was lower in RO (1.17) compared with other specialties (0.79), and fewer RO physicians (3/18, 17%) versus physicians in other specialties (19/42, 45%) were experiencing burnout as defined by PFI. No (0/7) RO residents were experiencing burnout. Median fulfillment score was similar in RO (3.00) compared with other specialties (2.83), and physicians who met PFI fulfillment criteria were less likely to be experiencing burnout than those who did not (13% vs 60%). Additionally, physicians who were mindful (MAAS score ≥ 4) were less likely to be burned out versus physicians who were not (11% vs 53%). These data are summarized in Figure 2.

**Fig. 3:**
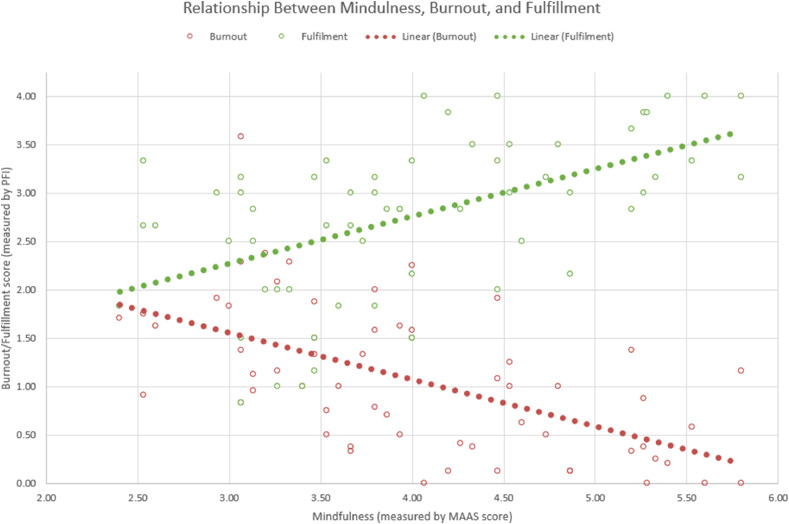
This figure consists of 2 superimposed scatter plots including a plot of mindfulness and burnout (red) and a plot of mindfulness and fulfilment (green). Burnout scores trended downward with increased mindfulness while fulfillment scores trended upward with increased mindfulness. Linear lines of best fit are included to aid in the visualization of the trend. Burnout and fulfillment scores were assessed using the professional fulfillment index (PFI). Mindfulness score was assessed using the mindfulness, attention, and awareness scale (MAAS).

On analysis with Pearson correlation coefficient, mindfulness (MAAS ≥ 4) positively correlated with fulfillment (*P* < .001, r = 0.534) and negatively correlated with both components of physician burnout, exhaustion (*P* < .001, r = -0.578) and disengagement (*P* < .001, r = -0.483). The relationship between mindfulness, fulfilment, and overall burnout (the average of exhaustion and disengagement scores) is shown in a scatter plot and linear lines of best fit in Figure 3. Univariate analysis for factors associated with burnout was significant for mindfulness (OR = 0.065, *P* < .001), RO versus other specialty (OR = 0.024, *P* = .044), working >60 hours per week (OR = 5.091, *P* = .018), spending >10 hours per week with family or friends (OR = 0.120, *P* = .001), and fulfillment (OR = 0.103, *P* < .001). Factors significant on univariate analysis were entered into a multivariate analysis, which found that mindfulness (OR = 0.123, *P* = .021) and fulfillment (OR = 0.179, *P* = .039) were associated with decreased risk of burnout. Table 2 displays the results of univariate and multivariate analyses.

**Table 2:** Univariate and multivariate analyses for factors associated with burnout

Univariate analysis		
Factor	Odds ratio for burnout (95% CI)	*P*
Mindfulness	0.065 (.013-.321)	< .001*
Debt more than $100,000	0.527 (.482-4.166)	.527
Debt more than $300,000	0.813 (.254-2.595)	.726
Income more than $300,000	0.980 (.329-2.915)	.97
Radiation oncology specialty vs other	0.242 (.061-.963)	.044*
Sleep 7 or more hours	1.605 (0.555-4.642)	.383
Weekly exercise	0.462 (0.157-1.359)	.161
Married	1.784 (.616-5.169)	.286
Having children	0.920 (.285-2.969)	.89
Working more than 60 hours a week	5.091 (1.319-19.649)	.018*
Spending more than 10 hours with family/friends	0.120 (.035-.405)	.001*
Fulfillment score	0.103 (.028-.369)	< .001*
Multivariate analysis		
Factor	Odds ratio for burnout (95% CI)	*P*
Mindfulness	0.123 (.021-.731)	.021*
Radiation oncology specialty vs other	1.135 (.039-32.615)	.941
Working more than 60 hours a week	3.657 (.688-19.456)	.128
Spending more than 10 hours with family/friends	0.446 (.084-2.383)	.345
Fulfillment score	0.179 (.035-.914)	.039*

*Abbreviation:* CI = confidence interval.

⁎ Statistically Significant.

### Discussion

To our knowledge this is the first study evaluating the role of mindfulness and professional fulfilment in burnout in a population including a significant proportion of RO professionals (30% including attendings and residents). We found that burnout was less common in RO residents and attendings relative to residents in other specialties at the same tertiary care academic center. Physicians with high levels of mindfulness and professional fulfilment had lower odds of burnout as assessed by multivariate analysis. Our univariate analysis indicated certain lifestyle factors such as specializing in RO, spending at least 10 hours per week with family and friends, and keeping weekly work hours to less than 60 were associated with lower odds of burnout, but this association did not maintain statistical significance on multivariate analysis.

### Burnout in RO

Our finding that physicians in our RO department were less burned out than residents in other specialties is consistent with other multispecialty assessments of burnout that have reported RO professionals to have less burnout relative to physicians in other specialties.^2^^,^^7^ The burnout rate (17%) among the 18 RO professionals in our study fell within the widely variant range of burnout rates (0%-49.5%) among RO specific populations in other studies.^8^^,^^10^^,^^11^^,^^20^, ^21^, ^22^ Comparing burnout rates between different studies is difficult due to differing burnout detection tools and varying populations studied. When the residents in our study were analyzed separately from the attendings, they had an abnormally low rate of burnout (0%, n = 7) relative to other resident populations. In comparison, a study surveying 232 U.S. RO residents by Ramey et al^8^ found that 33% experienced burnout, defined as either high emotional exhaustion or depersonalization on Maslach's burnout inventory (MBI). When investigating the relationship between resilience and burnout among Canadian RO residents using the same burnout criteria, Dahn et al^9^ found 42% of residents met criteria for burnout. Similar rates using the same criteria were present among RO residents in New Zealand and Australia (49.5%) and France (46%).

In contrast to the abnormally low rate among residents, burnout among attendings (27%) at our institution was higher than that of the residents and other reports of RO attending burnout in the literature, but still low compared with rates among attendings in other specialties.^2^^,^^7^^,^^23^ The discordance between attending and resident rates of burnout in this study are likely due to fundamental professional, financial, cultural, and social differences between residents and attendings. For example, relative to residents, attendings accept elevated professional and ethical responsibilities for the decisions they make on a daily basis, which could increase stress and feelings of inadequacy after bad clinical outcomes in their patients.

In contrast to our findings, the burnout rates among RO residents tend to be higher than those among RO attendings when comparing across studies in the literature,^8^^,^^9^^,^^11^^,^^20^^,^^22^ which may be related to less sensitive burnout criteria used by studies investigating RO attending burnout relative to those investigating RO resident burnout. A study by Kusano et al^11^ evaluating burnout among RO academic chairs found that they were generally less burned out than chairs in other specialties, and none met high burnout criteria, which in that study was defined as high emotional exhaustion, high depersonalization, and low accomplishment as assessed by MBI, a less sensitive threshold than used in the studies evaluating residents, which defined burnout as either high emotional exhaustion or depersonalization.^8^^,^^9^^,^^20^^,^^22^ Because 25% of chairs were at high risk for emotional exhaustion and 10% were at high risk for depersonalization, many would have met burnout criteria as assessed by the studies in the RO resident population. A study of burnout among RO residency program directors found only 6% to have high burnout using the same criteria as Kusano et al,^10^, ^11^ although 28% had high risk for emotional exhaustion and 15% had high risk for depersonalization. Selected studies of burnout are summarized in Table 3.

**Table 3:** Results of selected studies reporting burnout

Author	Population	Burnout assessment tool	Burnout rate among RO physicians	Burnout rate among all physicians included
Trockel et al^12^	Multispecialty	PFI*	N/A	39%
Trockel et al^12^	Multispecialty	MBI definition 1†	N/A	49%
Dahn et al^9^	Canadian RO residents	MBI definition 1†	42%	42%
Ramey et al^8^	US RO residents	MBI definition 1†	33%	33%
Kusano et al^11^	RO academic chairs	MBI definition 2‡	0%	0%
Aggarwal et al^10^	RO academic program directors	MBI definition 2‡	6%	6%
Shanafelt^2^ et al	Multispecialty	MBI definition 1†	41%	44%
Present study	Multispecialty	PFI*	17%	45%

*Abbreviations:* MBI = Maslach's burnout inventory; PFI = professional fulfillment index; RO = radiation oncology; US = United States.

⁎ Burnout = burnout score ≥ 1.33.

† Burnout = high score on either emotional exhaustion or depersonalization subscales.

‡ Burnout = high score on emotional exhaustion subscale, high score on depersonalization subscale, and low score on personal accomplishment subscale.

### Mindfulness and burnout in RO

We were unable to find previously reported data on mindfulness being protective against burnout in RO professionals, but our finding that more mindful physicians were less likely to experience burnout is consistent with reports in other specialties. In a study of an intervention of mindfulness training among residents in family medicine, anesthesia, and psychiatry, residents with higher MAAS scores were less likely to report high levels of residency-related stress, which was correlated with higher burnout scores.^13^ In addition to baseline mindfulness predicting burnout risk, mindfulness interventions decreased burnout level in both residents and attendings. Schroeder et al^24^ investigated a 13-hour weekend mindfulness training in a randomized study of 33 primary care attending physicians and found that physicians randomized to the mindfulness group showed improvements in MAAS score, stress, emotional exhaustion, and depersonalization 3 months after the intervention relative to baseline, a finding not present in the control group. A cohort study of 93 health care providers including 51 physicians found that an intervention of 2.5 hours of weekly mindfulness education for 8 weeks improved scores on all subsections of the MBI.^25^ A similar regimen was found to improve scores in all subsections of the MBI in a study of only primary care physicians.^15^

### Fulfillment and Work Exhaustion (WE)

Although focusing on developing mindfulness is helpful in the prevention of burnout, our data and others also suggest finding fulfilment at work, working moderate hours, and spending time with family can affect burnout risk. Professional fulfillment as measured by the PFI is a relatively new measure, but it has potential to capture many of the sources of well-being omitted in other popular assessments, such as happiness at work, sense of meaning at work, and professional self-sufficiency. The index was developed and validated by Trockel et al^12^ in response to an observation that widely used measures of physician well-being, most notably the MBI, lacked adequate assessments of these healthy characteristics of work life while focusing predominantly on burnout. Trockel et al^12^ found professional fulfillment to be significantly correlated with sleep-related impairment, depression, anxiety, and quality of life, all of which were also correlated with burnout. In a subsequent study assessing the association between burnout, fulfillment, and leadership, effective leadership positively correlated with fulfillment as assessed by PFI.^26^

The association in our study between work hours and burnout was mirrored in other studies of multidisciplinary and RO-specific populations. A large multispecialty study surveying 5197 physicians found each additional hour worked weekly to significantly increase the risk (OR, 1.02) of being burnt out on MBI.^2^ Similar findings have also been documented in RO-specific physician populations. Ramey et al^8^ found RO residents more likely to be experiencing burnout if they were working >60 hours weekly, and Kusano et al^11^ suggested that fewer hours spent at work may lower burnout in a population of RO chairs.

### Strengths, limitations, and future directions

There were limitations in our study. It was conducted at a single tertiary academic center and therefore fails to encompass the diversity of work culture, socioeconomic circumstances, and personnel variation present throughout the world. Our study also had a small sample size of respondents, which limited our ability to detect significance in the variables we evaluated. Additionally, fundamental professional, financial, and social differences exist between residents and attendings, and thus the inclusion of RO attendings in the comparison between RO physicians and residents in other specialties could be viewed as an unbalanced comparison. Because the attendings in the study experienced more burnout than the RO residents, we feel that the finding of relatively less burnout among RO physicians relative to residents in other specialties is likely to reflect a specialty-specific difference rather than confounding due to the inclusion of attendings. Third, survey distribution outside of the RO department was done by first sending the surveys to chief residents who then forwarded the surveys to their respective programs, so we do not know how many residents actually received the survey. Therefore, we cannot assess the response rate of the surveys. Fourth, systematic surveys are observational studies and can assess association, not causation, so our data alone do not definitively indicate that becoming more mindful will decrease burnout, although we hypothesize that it will based on interventions in other medical specialties.^15^ Finally, although the PFI is an experimentally validated instrument, all prior studies of burnout among RO professionals used MBI, which makes comparing our findings to the literature more difficult.

Despite its limitations, our study serves as the first suggestion that mindfulness and professional fulfilment could be protective against burnout in a physician population with significant RO representation. It furthermore demonstrates a precedent for using the PFI, an instrument that has strengths relative to the MBI. Specifically, the PFI has the ability to detect short-term responses to interventions, in contrast to many of the questions in the MBI, which study a timeframe of 12 months.^12^^,^^27^ The PFI also offers a thorough assessment of professional fulfilment, while the MBI does not assess positive aspects of employment besides professional achievement.^27^ Having identified the potential value of mindfulness for RO physicians using a scale designed for assessing interventions, our study sets the stage for the assessment of mindfulness practice as a way to ameliorate burnout in RO. Because our findings suggest that low fulfillment is a predictor of burnout, RO would benefit from investigating determinants of fulfillment.

Future mindfulness-based intervention studies in RO may incorporate multiple forms of mindfulness practices, the most common of which include focused attention, open monitoring, loving-kindness, and compassion mediations. Both focused attention and open monitoring are attention-based practices aimed at improving one's ability to control the way in which the practitioner directs attention, while compassion and loving-kindness are both constructive practices focused on cultivating healthy, adaptive feelings such as empathy and compassion.^28^ Given the demands of RO practice, an ideal mindfulness regimen for our profession might incorporate elements of all of the above mindfulness interventions. Facilitating attention management would help physicians work more efficiently and effectively, and cultivating empathy with our patients and colleagues may help improve work relationships and foster a sense of meaning at work.

## Conclusions

In summary, RO physicians experienced less burnout than residents in other specialties at our institution. Mindfulness, professional fulfillment, moderate work hours, and spending time with loved ones were associated with lower odds of burnout. In the future, studying mindfulness practice as an intervention and fulfillment as an important mental health endpoint will help us understand how best to improve the mental and emotional health of RO professionals.
